# Awareness, access to and uptake of HIV prevention interventions among youth in Zimbabwe: a population-based survey

**DOI:** 10.1186/s12879-025-11076-1

**Published:** 2025-05-16

**Authors:** Sophie H. Kelly, Steven Azizi, Chido Dziva Chikwari, Mandikudza Tembo, Tsitsi Bandason, Ethel Dauya, Constancia V. Mavodza, Tsitsi Apollo, Owen Mugurungi, Rashida A. Ferrand, Victoria Simms

**Affiliations:** 1 Department of Medicine, University of Cambridge, Cambridge, UK; 2 Department of Infectious Disease Epidemiology, London School of Hygiene & Tropical Medicine, London, UK; 3 Biomedical Research and Training Institute, Harare, Zimbabwe; 4 Department of Public Health, Environments and Society, London School of Hygiene & Tropical Medicine, London, UK; 5 AIDS and TB Unit, Ministry of Health and Child Care, Harare, Zimbabwe; 6 Clinical Research Department, London School of Hygiene & Tropical Medicine, London, UK

**Keywords:** Pre-exposure prophylaxis, Post-exposure prophylaxis, Voluntary medical male circumcision, Condoms, HIV prevention, Youth

## Abstract

**Background:**

Youth in southern Africa continue to be at high risk of HIV infection. We investigated the awareness of, access to, and uptake of HIV prevention interventions (pre-exposure prophylaxis (PrEP), post-exposure prophylaxis (PEP), voluntary medical male circumcision and condoms) among youth in Zimbabwe.

**Methods:**

A population-based survey of youth aged 18–24 years in 24 communities across three provinces was conducted between October 2021 and June 2022. An interviewer-administered questionnaire collected sociodemographic and sexual behaviour data including awareness of, access to, and use of HIV preventative interventions. Data were analysed using descriptive statistics and mixed-effects logistic regression weighted for clustering.

**Results:**

We recruited 17,682 youth (60.8% female, median age 20 years (Interquartile range 19–22)). Altogether 46.8% (*n* = 3634) of unmarried youth and 5.6% (*n* = 3538) of married youth reported consistent condom use and 49.8% (*n* = 3369) of men reported being circumcised. Awareness of PrEP and PEP was 11.2% and 11.9% respectively. 6900 participants (38.4%) reported at least one eligibility criterion for PrEP. Eligibiltiy criteria included having multiple partners or receiving money or goods for sex in the last year, HIV-negative individuals in serodiscordant relationships, those who had ever been treated for an STI, ever injected drugs, been pregnant or taken PEP. In comparison to the non-eligible population (*n* = 10782), the eligible population were more likely to have heard of PrEP (13.5% vs. 9.9%, *p* < 0.001), been offered PrEP if they had heard of it (17.0% vs. 6.3%, *p* < 0.001) and to have ever taken PrEP if offered it (60.7% vs. 27.0%, *p* < 0.001). Those in the richest wealth quintiles and with higher education level were more likely to have heard of PrEP and report regular condom use. Forty-two of 199 (20.2%) who reported having experienced forced sex in the last year sought healthcare afterwards, of these 17 of 42 (36.0%) had been offered PEP and 12 of 17 (63.7%) had ever taken it.

**Conclusions:**

Use of HIV preventive interventions remains limited among youth despite longstanding HIV programmes. Lack of awareness limits use of PrEP and PEP. There are underlying socioeconomic barriers limiting awareness of and demand for condoms, circumcision and pharmacological prophylaxis. These must be urgently addressed.

**Trial registration number:**

NCT03719521.

**Supplementary Information:**

The online version contains supplementary material available at 10.1186/s12879-025-11076-1.

## Background

Approximately 39 million people were living with HIV globally in 2022, over 50% in east and southern Africa [1]. While there has been a general global decline in HIV infections due to the development and scale-up of a variety of effective HIV prevention methods, the decline in incidence has been much less in youth compared to other age-groups [1, 2]. In 2019, globally two out of every seven new HIV infections were among people aged 15–24 years [3]. In southern Africa, young women are at particularly high risk and are three times more likely than their male counterparts to acquire HIV [1]. If the global commitment to end HIV as a public health threat by 2030 is to be achieved, youth need to be a priority group for HIV prevention programmes [4].

The 2025 UNAIDS HIV prevention road map recommends the scale-up of HIV combination prevention measures such as (male and female) condoms and antiretroviral based primary prevention, including pre-exposure prophylaxis (PrEP) and post exposure prophylaxis (PEP), among key populations and young people [5].

Youth face specific social, personal, structural and economic barriers to access of HIV prevention services and adopting lower risk sexual behaviours [6, 7]. Understanding utilisation of prevention approaches in youth will be critical to deciding on where best to direct resources to address gaps and thus to meet 2030 sustainable development goals to eliminate HIV as a public health threat [8]. We investigated the awareness of, access to, and uptake of key preventative measures namely condom use, voluntary male medical circumcision (VMMC), PrEP and PEP among youth in Zimbabwe.

## Methods

### Study design

Data for this study was obtained from a population-based survey, conducted between October 2021 and June 2022 to ascertain the outcomes of a cluster randomised trial (CHIEDZA) investigating the impact of community-based integrated HIV and sexual and reproductive health services for youth on population-level HIV outcomes (Trial Registration Number: NCT03719521).

The trial protocol including details of the intervention and the outcome survey has been published elsewhere [9]. Briefly, the trial was conducted across three provinces of Zimbabwe: Harare, Bulawayo, and Mashonaland East. Eight clusters (geographically demarcated areas with a community centre from where the intervention could be delivered, and a primary care clinic) in each province (total 24 clusters) were delineated and randomised 1:1 (stratified by province) to the intervention (HIV and sexual and reproductive health services delivered by a multi-disciplinary team) or to the control (existing health services) arm. Following 30 months of intervention delivery, a population-based survey of 700 youth aged 18–24 years per cluster (total target *n* = 16800) was undertaken to ascertain trial outcomes.

### Population-based survey

Each cluster was mapped and divided into sections of road which were randomly sampled. All households in the selected road sections were enumerated, and all eligible individuals (defined as those aged 18–24 years and resident in the household) were invited to participate.

An interviewer-administered questionnaire was used to collect sociodemographic data including age, sex, occupation, marital status, education, household income and asset ownership. Wealth quintiles were calculated using a composite of household income and asset ownership relative to the local population. Participants were defined as “married” if they reported “being married” or were “living as married”. Data on past and current pregnancy and sexual behaviour including whether participants had ever had intercourse, their number of sexual partners, history of HIV testing, testing and treatment of sexually transmitted infections (STIs) and use of HIV prevention interventions was collected (Supplementary Table 1).

The frequency of male condom use, either personal or by partner, and whether condoms had been used at the last sexual encounter was recorded. In addition, women were asked whether they were currently using female condoms as a method of contraception and whether they had used them in the last 12 months. The term ‘condom’ without qualifier is used to refer to the male condom hereafter. Participants who reported condom use “most of the time” were coded as having consistent condom use.

All participants were asked about whether they had ever heard of drugs to prevent HIV before possible exposure (PrEP), whether they had ever been offered PrEP by a clinic or organisation and if they ever taken PrEP. They were asked if they ever heard of drugs to prevent HIV after a possible exposure (PEP) and if they had ever taken PEP. Participants were asked about whether they had ever experienced forced sex, if so, how many episodes they had experienced in the last 12 months, whether they had accessed health care after a sexual assault in the last year, what kind of healthcare facility they had attended and whether PEP had been offered.

Males were asked if they had ever been offered or referred for VMMC by a clinic, school or VMMC programme, and whether they were circumcised. Men who reported being circumcised were asked at what age they had been circumcised and the reasons for undergoing circumcision. If uncircumcised they were asked whether they knew where they could get circumcised, how likely they would be to get circumcised if it was free and easily available, and their reasons for not being circumcised.

Participants were asked about their history of HIV testing, knowledge of their own HIV status, and a dried blood spot sample was taken for pseudoanonymised HIV testing. Samples were tested for the presence of antiretroviral drugs (ARVs) if they were positive for HIV and the viral load was below 10,000 copies/ml, but the participant had not self-reported as HIV positive.

### Eligibility for HIV prevention interventions

Recognising that certain prevention methods are available for specific populations; we used Zimbabwe Ministry of Health and Child Care (MoHCC) guidance to inform our eligible population for each prevention method. For condoms, the eligible population was all sexually active youth. The eligible population for VMMC was all male youth.

The MoHCC recommends PrEP should be provided to those “*at substantial risk of HIV infection*” and specifies that this includes adolescent girls and young women, male and female sex workers, HIV negative people in a serodiscordant relationship, men who have sex with men and transgender women, pregnant and lactating mothers, those who have ever taken PEP, people who inject drugs and all persons who perceive themselves at high risk of HIV infection including those with multiple sexual partners and those who have had an STI [10]. Based on this, those considered eligible for PrEP in this study included those who reported more than one partner in the last 12 months, those who had received money or goods for sex in the last 12 months, those who were known to be HIV negative in a serodiscordant relationship, those who had ever been treated for an STI, those who had ever injected drugs, women who had ever been pregnant, and those who had ever taken PEP.

The MoHCC recommends PEP should be available to all those who have an occupational exposure to HIV, those who have experienced sexual assault or following unprotected sex [10]. Within our cohort we focused on PEP awareness and uptake among youth who reported forced sex.

### Data analysis

Data were analysed in R Studio (Version 4.2) using cluster methods weighted to account for clustering by trial cluster and province. Multivariable mixed effects logistic regression models were used to determine factors associated with awareness, access, and uptake of PrEP and condom use, with both cluster and province included as random effects. This approach accounted for the inherent hierarchical structure of the data. Age, sex, education status, wealth quintile, formal employment were included as fixed effects in all models. Interaction terms for wealth quintile, education and employment were tested for improved fit using analysis of variance. The independent binary variables included in mixed effects models for PrEP awareness, offer and uptake were having more than one partner in the last 12 months, known to be HIV negative in a serodiscordant relationship, ever being treated for an STI, ever been paid for sex, ever been pregnant, and ever having taken PEP. In the model for condom use, marital status was the independent binary variable. We describe the missing data for each variable. Participants with missing data in the model variables described above were excluded from the relevant model. Statistical significance was defined as a p-value less than 0.05.

## Results

### Study population

The study enumerated 18,727 individuals, of which 18,539 (98.9%) were eligible and 17,682 (95.3%) were surveyed; median age 20 (IQR 19–22) years, 39.3% (6940) male. Demographic characteristics are shown in Table 1. Of note, 1226 people were categorised as living with HIV based on a positive DBS result (*n* = 1200) or self report. Of these, 650 participants (53.0%) either self-reported living with HIV or had anti-retroviral drugs detected in their blood sample.

**Table 1:** Baseline demographic and sexual behavioural characteristics of study population

Characteristic			*N*	Missing *N* (%)	Female, *N* = 10,741 (59^1^%)	Male, *N* = 6940 (41%)
**Age (years); median (IQR)** ^**2**^		17,681		0 (0)	20 (19.0, 23.0)	20 (18.0, 22.0)
**Province**		17,681		0 (0)		
Bulawayo					3373 (33.0%)	2596 (37.6%)
Harare					3905 (19.4%)	1944 (14.5%)
Mashonaland East					3463 (47.5%)	2400 (47.9%)
**Maximum level of education**		17,681		0 (0)		
Primary school					2319 (22.7%)	935 (14.2%)
Secondary school					7651 (70.9%)	5365 (77.2%)
Post-secondary					771 (6.5%)	640 (8.6%)
**Currently in formal employment or education**		17,681		0 (0)		
Yes					2948 (26.8%)	2849 (39.6%)
No					7793 (73.2%)	4091 (60.4%)
**Ever had sexual intercourse**		17,662		89 (< 0.01)		
Yes					7029 (65.2%)	4263 (60.6%)
No					3670 (34.5%)	2630 (38.8%)
**Age of first sexual intercourse (years); median (IQR)** ^**2**^		11,125		167 (0.02)	18 (17.0, 19.0)	17 (16.0, 18.0)
**Marital Status**		17,681		0 (0)		
Yes					3272 (30.6%)	287 (4.4%)
No					7469 (69.4%)	6653 (95.6%)
**More than one lifetime partner**		17,201		480 (2.7)		
Yes					3200 (30.4%)	3314 (49.2%)
No					7367 (69.6%)	3320 (50.8%)
**More than one partner in last 12 months**		17,453		228 (1.4)		
Yes					735 (6.7%)	1872 (26.7%)
No					9912 (93.3%)	4934 (73.3%)
**Ever had an HIV test**		17,635		46 (0.2)		
Yes					7960 (73.9%)	4193 (59.5%)
No					2766 (26.1%)	2716 (40.5%)
**Know the results of HIV test**		12,153		0 (0)		
Yes					7864 (98.7%)	4138 (98.6%)
No					96 (1.3%)	55 (1.4%)
**Ever had an STI**		17,573		108 (0.6)		
Yes					573 (5.0%)	258 (3.5%)
No					10,116 (95.0%)	6626 (96.5%)
**HIV negative in a serodiscordant relationship**		17,681		0 (0)		
Yes					25 (0.2%)	5 (0.1%)
No					8313 (77.7%)	5119 (73.4%)
Unknown status of participant or partner					2403 (22.1%)	1816 (26.6%)
**Known HIV positive status (self-report)**		17,681		0, 0		
Yes					368 (3.4%)	67 (0.9%)
No					10,373 (96.6%)	6873 (99.1%)
**Known HIV positive status (self-report or ARV detection)**		17,681		1 (< 0.01)	487 (4.5%)	163 (2.2%)
Yes					10,254 (95.5%)	6777 (97.8%)
No						
**Received payment for sex in last 12 months**		17,455		226 (1.4)		
Yes					163 (1.5%)	87 (1.3%)
No					10,481 (98.5%)	6710 (98.5%)
Didn’t say					4 (0.0%)	10 (0.2%)
**Paid for sex in last 12 months**		17,455		226 (1.4)		
Yes					41 (0.4%)	154 (2.2%)
No					10,601 (99.6%)	6,645 (97.6%)
Didn’t want to say					6 (0.0%)	8 (0.1%)
**Ever been forced to have sex**		17,609		72 (0.4)		
Yes					219 (1.9%)	53 (0.7%)
No					10,484 (98.1%)	6,853 (99.3%)
**Ever used injected drugs**		17,627		54 (0.3)		
No					10,722 (100.0%)	6,897 (99.9%)
Yes					2 (0.0%)	6 (0.1%)
**Ever taken PEP**		17,622		59 (0.3)		
Yes					71 (0.6%)	40 (0.5%)
No					10,637 (99.4%)	6,874 (99.5%)
**Ever been pregnant**		10,729				
Yes				13 (0.1)	4,037 (40.4%)	NA
No					6,422 (59.6%)	NA

^1^Percentage weighted for clustering

^2^Interquartile Range

### Missing data

Overall, there were low levels of missing data (Table 1). Eighty-nine people (0.5%) did not answer the question on whether they had ever had intercourse, 480 people (2.8%) who had had sex did not answer the question on number of lifetime partners and 228 (1.4%) did not provide an answer to the number of partners in the last 12 months. Participants who responded that they did not want to say whether they have ever had sex were not asked subsequent questions about sexual partners and behaviours over the last 12 months. Overall, 592 participants (3.3%) did not respond to one or more specific questions about their sexual behavioural characteristics including whether they had ever had an HIV test, ever had an STI test, ever had STI treatment, ever had intercourse, ever paid for sex, ever received payment or goods for sex, their number of partners in last 12 months, number of lifetime partners, whether they had ever heard of PrEP, ever heard of PEP or ever taken PEP. Participants with missing data were more often male, from Bulawayo and more frequently reported having had an STI, high levels of alcohol use, ever having used recreational drugs, transactional sex or forced sex (Supplementary Table 2).

### Eligible groups

The population for condom use consisted of 11,293 (63.6%) people who reported ever having sexual intercourse, of whom 7755 (68.7%) were not married. Overall, 6900 of 17,682 (38.4%) met the eligibility criteria for PrEP: 2607 of 17,682 people (14.7%) reported more than one partner in the last 12 months, 4037 of 10,023 women (40%) reported ever being pregnant, 831 of 17,681 (4.4%) had ever been treated for an STI, 30 (0.2%) reported being HIV negative in serodiscordant relationships, 250 participants (1.5%) reported having ever received payment or goods for sex (163 female, 87 male), 111 participants (0.5%) reported having ever taken PEP and 8 participants (0.1%) reported having ever injected drugs. There was some overlap between the groups (Supplementary Fig. 1). Two-hundred and seventy-two participants (1.4% of the population, 1.9% of the women and 0.7% of the men) reported having been forced to have sex and were included in our eligible population for PEP.

### Condom use

Overall, 3833 of 11,293 (33.9%) of sexually active youth reported consistent condom usage and 4459 of 11,293 (39.5%) reported condom use during their last sexual encounter (Table 2). Sixteen women reported currently using female condoms for contraception and 36 reported using them in the last 12 months. Among sexually active youth, the proportion reporting that they or their sexual partner(s) consistently used condoms and that they or their partner used a condom during last sexual encounter was lower among females compared to males (22.2% vs. 52.2% and 26.6% vs. 60.3% respectively *p* < 0.001) (Table 2). Condom use among sexually active married youth was particularly low; 199 of 3538 (5.6%) of married youth reported consistent condom usage and 227 of 3538 (6.3%) reported condom usage in last sexual encounter. Seven of twenty-seven (29.1%) HIV negative partners in a known serodiscordant relationship and 135 of 343 (40.3%) of youth who self-reported living with HIV reported consistent condom use. In a mixed effects logistic regression model, consistent condom use was negatively associated with being married (Odds Ratio (OR) = 0.09, 95% confidence interval (CI) 0.08–0.11), being female (OR = 0.42, 95% CI 0.38–0.47) and significantly associated with education status (χ² = 10.1, *p* = 0.006) and wealth quintile (χ² = 17.1, *p* = 0.001) with those in the richest wealth quintile 19% more likely to report condom use than those in the poorest wealth quintile (OR = 1.19, 95% CI 1.00–1.41) and those with post-secondary education 24% more likely to report consistent condom usage that those with primary education only (OR = 1.24, 95% CI 1.01–1.52) (Supplementary Table 3).

**Table 2:** Reported consistent condom usage and condom use during last sexual encounter among those reporting they had ever had sex by sociodemographic factors

Characteristic	Overall, *N* = 11,293	Consistent Condom Use over last 12 months, *N* = 3833 (34%)^1^	Condom Use Last sexual encounter, *N* = 4459 (40%)	
**Sex**				
Female	7029	1582 (22.2%)		1897 (26.6%)
Male	4263	2251 (52.2%)		2562 (60.3%)
**Age Group**				
18–20	4361	1720 (39.2%)		2008 (46.2%)
21–24	6932	2113 (30.5%)		2451 (35.6%)
**Province**				
Bulawayo	4001	1818 (45.2%)		2073 (51.6%)
Harare	3806	1036 (27.6%)		1211 (32.4%)
Mash East	3486	979 (27.2%)		1175 (33.1%)
**Married**				
Yes	3538	199 (5.6%)		227 (6.3%)
No	7755	3634 (46.8%)		4232 (55.1%)
**Level of highest education**				
Primary school	2302	531 (22.1%)		655 (27.9%)
Secondary school	8000	2869 (36.2%)		3300 (41.8%)
Post secondary	991	433 (45.2%)		504 (53.2%)
**In Formal Employment or Education**				
Yes	2749	1325 (49.0%)		1558 (58.1%)
No	8544	2508 (29.3%)		2901 (34.2%)
**Wealth quintile**				
Poorest	2603	555 (21.6%)		636 (24.9%)
Poor	2286	655 (29.1%)		769 (34.5%)
Medium	2307	835 (37.0%)		993 (43.7%)
Rich	2082	874 (42.8%)		1011 (50.1%)
Richest	2002	911 (46.6%)		1047 (54.6%)
**Ever treated for STI** ^**2**^				
Yes	831	247 (29.1%)		301 (36.1%)
No	10,443	3578 (34.3%)		4148 (40.0%)
**HIV negative in a serodiscordant relationship**				
Yes	27	7 (29.1%)		8 (31.7%)
No	7082	2272 (31.7%)		2639 (37.1%)
Unknown status of participant or partner	4184	1554 (37.6%)		1812 (44.3%)
**Known HIV positive status (self-report)**				
Yes	343	136 (40.3%)		171 (50.6%)
No	10,950	3697 (33.7%)		4288 (39.4%)
**Known HIV positive status (self-report or ARV detection)**				
Yes	474	195 (40.9%)		250 (52.0%)
No	10,819	3638 (33.6%)		4209 (39.2%)
**More than one partner in last 12 months**				
Yes	2608	1484 (57.7%)		1682 (66.1%)
No	8546	2348 (27.3%)		2777 (32.5%)
**Received payment or goods for sex in last 12 months**				
Yes	250	120 (46.1%)		142 (56.6%)
No	10,073	3706 (36.8%)		4311 (43.1%)
**Paid for sex in last 12 months**				
Yes	195	120 (60.6%)		136 (70.6%)
No	10,128	3706 (36.6%)		4316 (42.9%)

^1^ Percentages weighted for clustering

^2^Sexually Transmitted Infection

Participants who had ever had intercourse were asked where they bought condoms. Of those who responded to the question (3271 of 7029 women (46.5%) and 3304 of 4263 men (77.5%)), men more frequently reported obtaining condoms from informal settings than women; for example, supermarkets or kiosks (36.7% vs. 27.0%, *p* < 0.001), family members or friends (20.9% vs. 6.6%, *p* < 0.001), or bottle stores (13.6% vs. 3.8% *p* < 0.001) (Supplementary Table 4). Of those who had ever had intercourse, 2503 of 7029 (35.6%) women and 1922 of 4263 (44.1%) men listed any barrier to obtaining condoms. The most common barriers to obtaining condoms were being too embarrassed to ask for condoms (65.4% of women who listed any barrier and 50.2% of men) and lack of privacy/confidentiality in places they would go for condoms (46.6% women and 41.7% men). Concerns about cost were frequently reported (21.1% of women and 36.2% men) as well as concerns about quality (19.7% of women and 26.9% of men). Concerns about limited opening hours of places where condoms are purchased, distance to travel and stockouts were less frequently reported (Supplementary Table 5).

### Voluntary medical male circumcision

Overall, 62.7%, 4355 of 6490 male participants reported being offered or referred for VMMC. Most men were referred through school-based programmes. Of those offered VMMC, 2898 of 4355 (68.6%) had been circumcised. The most common reason for circumcision cited by 75.6% of those circumcised was STI/HIV prevention (Supplementary Table 6).

A small number of males (*n* = 471) reported that they had been circumcised but had not been offered or referred for VMMC. Of these, 15.8% specified cultural reasons for circumcision compared to 8.9% of the 2898 of those offered and circumcised through a VMMC programme (*p* = 0.09). The median age of circumcision was 14 (IQR 12–16) years, with 3.0% of circumcised men reporting an age of circumcision younger than 10 years old and 3.7% reporting circumcision aged 20 years or older.

**Fig. 1:**
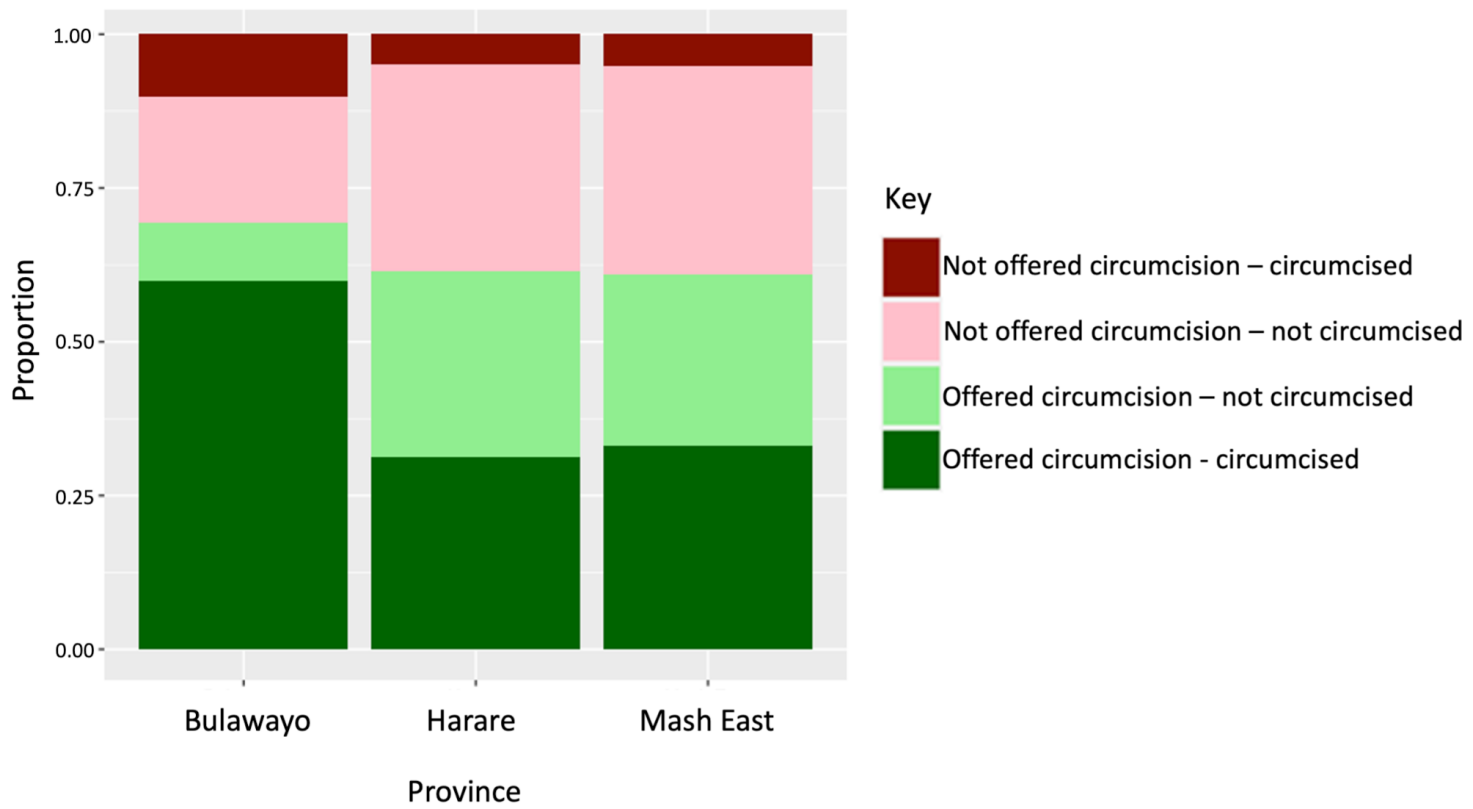
The proportion of males offered circumcision and who were circumcised across the three provinces surveyed

Figure 1 shows uptake of circumcision by province. Men in Bulawayo were significantly more likely to have been offered circumcision than in Harare or Mashonaland East (68.8% vs. 59.8% and 58.9% respectively, *p* = 0.026) and reported uptake was much higher in Bulawayo; 86.5% of those offered circumcision were circumcised vs. 50.9% of those in Harare and 54.3% of those in Mashonaland East. Of the 471 men who were circumcised outside of VMMC programmes, 262 (53.2%) were in Bulawayo, 91 (10.5%) were in Harare and 118 (36.2%) were in Mashonaland East.

Among the 3410 uncircumcised men, 1523 (44.5%) listed fear of pain as a reason for not being circumcised; 299 of the 3410 men (8.8%) cited never being offered circumcision as a reason they were not circumcised (Supplementary Table 7). Notably, 2145 of 3410 uncircumcised men (60.8%) knew how to access circumcision if they wanted it, and 1125 of 3410 uncircumcised men (32.9%) stated they would probably or definitely take up VMMC if it was free and easily available.

### Pharmacological prophylaxis

Two thousand and sixty (11.2%) and 2218 (11.9%) of 17,683 participants had heard of PrEP and PEP respectively (Table 3). Awareness was higher in those who were the most educated, most wealthy and those in eligible groups (Table 3). Eight people reported having ever injected drugs of whom 3 had heard of PrEP and none had taken PrEP. We did not analyse this group further due to its small size.

**Table 3:** Awareness of HIV Pre-Exposure prophylaxis and post exposure prophylaxis by sociodemographic and sexual behavioural characteristics

Characteristic	Total *N* = 17,641 (100%)	Heard of PrEP^1^, *N* = 2060 (11.2%^2^)	Heard of PEP *N* = 2218 (11.9%)
**Sex**			
Female	10,722	1317 (12.0%)	1392 (12.5%)
Male	6918	742 (10.2%)	825 (11.0%)
**Age Group**			
18–20	9235	862 (9.1%)	965 (10.0%)
21–24	8405	1198 (13.8%)	1253 (14.2%)
**Province**			
Bulawayo	5934	988 (16.5%)	1028 (17.3%)
Harare	5844	610 (10.9%)	707 (12.4%)
Mash East	5863	462 (7.6%)	483 (7.9%)
**Married or Living as Married**			
Yes	3558	314 (8.4%)	317 (8.5%)
No	14,083	1746 (12.0%)	1901 (12.8%)
**Level of highest education**			
Primary school	3250	251 (7.3%)	259 (8.0%)
Secondary School	12,983	1426 (10.7%)	1561 (11.4%)
Post-secondary	1407	383 (27.3%)	398 (27.8%)
**In formal employment or education**			
Yes	5780	899 (15.3%)	1002 (16.5%)
No	11,857	1161 (9.4%)	1216 (9.8%)
**Wealth quintile**			
Poorest	3650	285 (6.6%)	282 (6.6%)
Poor	3418	349 (9.8%)	381 (10.8%)
Medium	3575	390 (11.4%)	404 (11.4%)
Rich	3456	487 (14.7%)	525 (15.2%)
Richest	3522	547 (15.5%)	624 (17.5%)
**Ever treated for an STI**			
Yes, in the last year	550	109 (19.0%)	119 (21.2%)
Yes > 12 months ago	280	61 (21.7%)	67 (22.3%
No	16,727	1867 (10.8%)	200 (11.4%)
**Ever tested for an STI (not HIV)**			
Yes	2001	444 (22.9%)	478 (24.4%)
No	15,614	1611 (10.0%)	1735 (10.5%)
**Ever had an HIV test**			
Yes	12,138	1684 (13.4%)	1788 (14.0%)
No	5474	376 (6.9%)	430 (7.5%)
**HIV negative in a serodiscordant relationship**			
Yes	30	10 (33.6%)	6 (20.1%)
No	13,433	1571 (11.2%)	1717 (12.0%)
Unknown status of partner or participant	4220	479 (11.3%)	495 (11.6%)
**Known HIV positive status (self-report)**			
Yes	435	111 (26.4%)	110 (25.7%)
No	17,202	1949 (10.9%)	2108 (11.6%)
**Known HIV positive status (self-report or ARV detection)**			
Yes	650	138 (22.0%)	141(21.8%)
No	16,990	1922 (10.9%)	2077 (11.6%)
**More than one partner in last 12 months**			
Yes	2599	425 (15.6%)	443 (15.9%)
No	14,831	1592 (10.4%)	1721 (11.1%)
**Received payment or goods for sex in last 12 months**			
Yes	250	71 (24.9%)	69 (24.3%)
No	16,350	1850 (11.0%)	1981 (11.5%)
**Ever injected drugs**			
Yes	8	3 (24%)	4 (40%)
No	17,597	2053 (11.3%)	2210 (11.9%)
**Ever been pregnant**			
Yes	4305	524 (11.7%)	516 (11.5%)
No	6412	793 (12.6%)	876 (13.2%)

^1^Pre exposure prophylaxis

^2^Percentages weighted for clustering

**Fig. 2:**
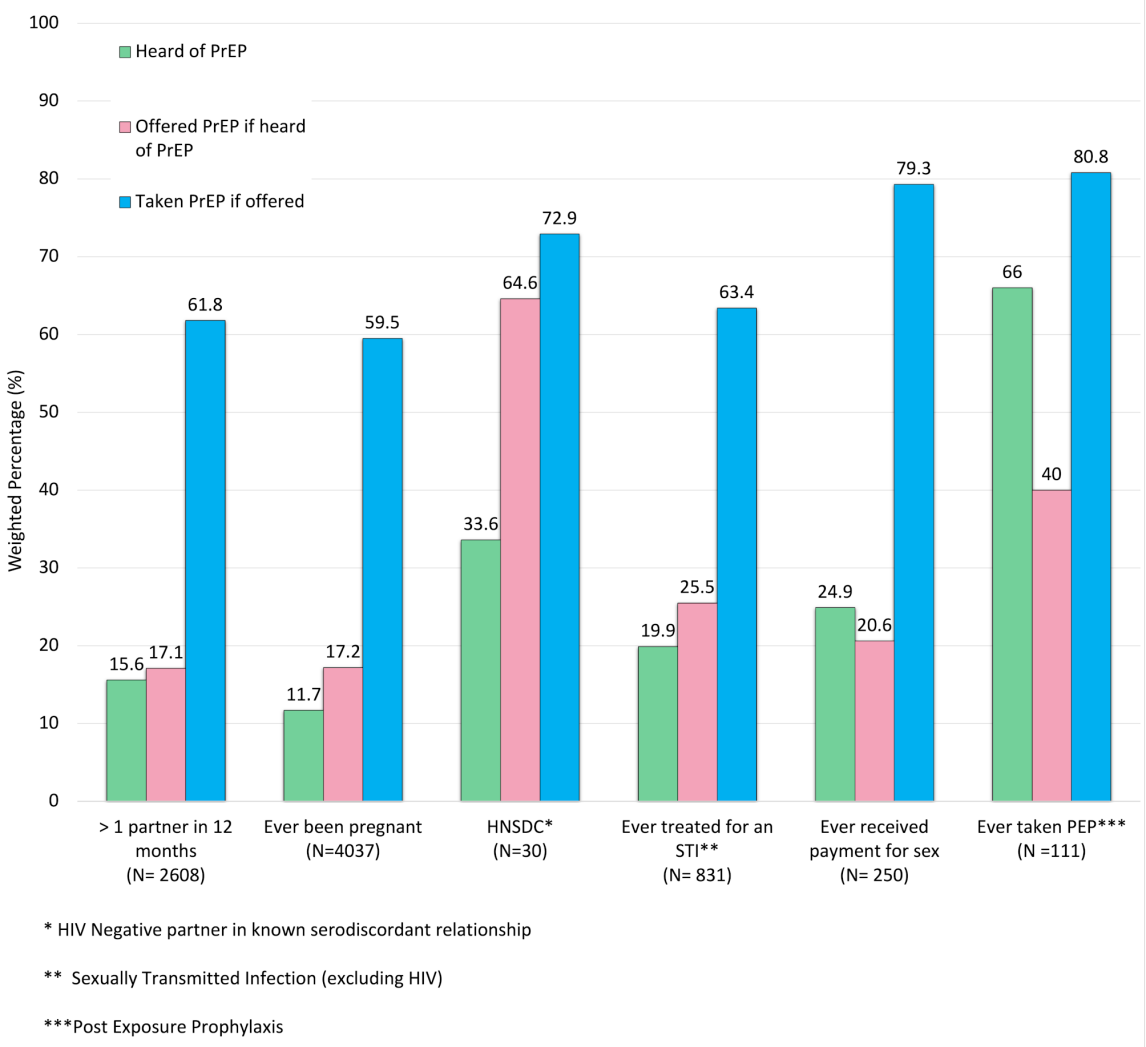
The proportion of people who have heard of, been offered and taken Pre-Exposure Prophylaxis (PrEP) across target populations

Participants in eligible groups (as defined above) were more likely to have heard of PrEP and to have been offered PrEP if they had heard of it and to take it if offered (Table 4). Uptake of PrEP was more than 50% when offered in all eligible populations (Fig. 2).

**Table 4:** The proportion of the population surveyed within CHIEDZA who have heard of, been offered and ever taken PrEP

Characteristic	Overall, *N* = 17,683 (100%)^1^	Non- Eligible Population *N* = 10,783 (62%)^1^	PrEP Eligible Population^4^ *N* = 6900 (38%)^1^	*p*-value^2^
Heard of PrEP (*N* = 17683)	2060 (11.3%)	1093 (9.9%)	967 (13.5%)	< 0.001
Offered PrEP if heard of PrEP (*N* = 2060)	235 (11.2%)	69 (6.3%)	166 (17.0%)	< 0.001
Ever taken PrEP if offered PrEP (*N* = 234)	118 (50.5%)	18 (27.0%)	100 (60.7%)	< 0.001

^1.^ Percentages weighted for clustering

^2.^ chi-squared test with Rao & Scott’s second-order correction

^3.^ Pre-exposure prophylaxis

^4.^ Participants meeting one or more of the following criteria: more than one partner in the last 12 months, ever been pregnant, ever been treated for an STI, received payment or goods for sex in the last 12 months, known to be HIV negative in a serodiscordant relationship, ever taken HIV post exposure prophylaxis.

A lower proportion of women who had ever been pregnant had heard of PrEP compared to sexually active women who had never been pregnant (11.8% vs. 16.7%, *p* < 0.001), but this was not statistically significant in the multivariable model adjusted for age, sex, wealth quintile, education status and employment status (OR = 0.88, 95% CI 0.75–1.03) (Table 5).

**Table 5:** Associations between high-risk exposures and PrEP awareness, access and uptake in multivariable mixed effects models adjusted for age, sex, wealth Quintile, education status and employment status as fixed variables, Province and cluster as random variables. Each exposure and outcome variable were analysed in individual models

Exposure	Heard of PrEP OR [95% CI]	Offered PrEP^a^ OR [95% CI]	Taken PrEP^b^ OR [95% CI]
**> 1 partner in last 12 m** ^**1**^	1.52 [1.34, 1.73]	1.84 [1.32,2.58]	2.09 [1.09, 4.00]
**Ever been pregnant** ^**2**^	0.88[0.75, 1.03]	1.18[0.77,1.82]	1.33[0.61,2.93]
**Known to be HIV Negative in Sero-discordant relationship** ^**3**^	4.71 [2.13,10.4]	7.24 [1.92,27.3]	2.29 [0.24,22.3]
**Ever treated for STI** ^**4**^	1.96 [1.63,2.36]	2.42 [1.62, 3.61]	1.88 [0.91, 3.89]
**Been paid for sex in last year** ^**5**^	3.00 [2.23,4.03]	1.93 [1.04,3.58]	3.23 [0.85,12.2]
**Ever taken PEP** ^**6**^	10.5 [6.94,15.9]	5.49 [3.16, 9.52]	4.44 [1.62, 12.2]
**Eligible for PrEP** ^**7**^	1.44 [1.30,1.60]	2.38 [1.71,3.30]	3.42 [1.74, 6.70]

^a)^ – offered PrEP if heard of PrEP

^b^ – Taken PrEP if offered PrEP

^1^Reference group is those who 1 or fewer partners in the last 12 months

^2^Reference group is sexually active women who have never been pregnant

^3^Reference group includes both who know they are not in a serodiscordant relationship and those with unknown status of either themselves or their partner

^4^Reference group is all participants who did not report being treated for an STI

^5^Reference group is all participants who did not report having received payment for sex in the last year

^6^Reference group is all participants who did not answer yes to any of the questions as above

^7^Reference group is all participants who were not eligible for PrEP

The majority of those who had been offered and had taken PrEP (100 of 118, 83.8%) reported one or more factor that put them at higher risk of HIV acquisition. Notably, 28 people reported having taken PrEP despite not being offered it from a clinic, likely buying it privately or acquiring it through social networks. Compared to those who obtained PrEP from a clinic, this group of 28 people were more likely to be in the richest quintile (50.5% vs. 16.7% *p* = 0.008), highly educated (22.0% with post-secondary education vs. 11.8%, *p* = 0.04), in education or formal employment (54.7% vs. 27.1%, *p* < 0.01) and unmarried (97.1% vs. 76.4%, *p* = 0.02).

Awareness of PEP was higher in those who had ever experienced forced sex compared to the general sample (25.7% vs. 11.7%, *p* < 0.001) and 21.0% of those who reported ever being forced to have sex reported having ever taken PEP compared to 4.0% of the whole cohort. Of the 272 participants who reported having been forced to have sex, 204 (68.3%) reported one or more episodes in the last year. Forty-two (20.2%) of those who had been forced to have sex in the last 12 months reported seeking healthcare; 22 attended a primary care clinic, 10 attended hospital, 9 attended a rape or sexual assault clinic, and 1 did not say. Of those who sought healthcare, 17 of 42 (36.0%) were offered PEP. Of participants who attended hospital 8 of 10 (79.0%) were offered PEP compared to 5 of 9 (52.0%) who attended specialist sexual assault clinics and 4 of 20 (17.0%) who attended primary healthcare clinics. Of the 17 people who reported being offered PEP after sexual assault in the last 12 months, 12 (63.7%) reported having ever taken PEP.

## Discussion

Our study found low overall usage of HIV prevention interventions including condoms, VMMC and pharmacological prophylaxis (PrEP and PEP) among youth compared to MoHCC targets. In addition, youth in lower socioeconomic quintiles and with lower levels of education had a disproportionate lack of access to and use of all preventative measures discussed.

Less than half of youth reported consistent condom use. Condom use was low particularly among females, in those who are married and those in the lowest wealth quintiles. The low levels of condom use among married individuals and the fact that only 53.0% of those living with HIV knew their status is of particular concern because as HIV epidemics have matured, nearly two-thirds of total HIV incidence in Africa occurs within the context of marriage or cohabitation [11, 12]. Of these, approximately half of infections are attributed to through extra-partner sexual encounters and the remainder occur through transmission within a couple [11]. Rates of HIV status disclosure by youth to their sexual partners, including regular sexual partners is low [13]; but those in stable relationships may incorrectly perceive themselves to be at lower risk of acquiring HIV. Given that in this study only 53% of people living with HIV knew their status, less than a third of HIV-negative individuals in HIV serodiscordant relationships and 40.9% of those who were known to be living with HIV used condoms regularly, the barriers to condom use need to be urgently addressed.

The association of being female with lower condom use is not surprising and is consistent in studies across the eastern and southern African region [14]. Studies have consistently reported the difficulties women face in negotiating safer sex in their relationships and that, in particular, women are apprehensive about the consequences of suggesting condoms use to their sexual partners [15]. The reasons are multifactorial including patriarchal societal structures, where women have an inferior social position within marital relationships and may have little decision-making power when negotiating sex either within stable or casual sexual partnerships [16]. This may be exacerbated by societal norms about appropriate sexual behaviour of males versus females whereby women who do ask for condoms may be perceived as overly interested in sex, distrustful of their male partners, or promiscuous [17]. Women may also fear the consequences of asking for condom use such as violence or abandonment especially where cultural practice such as bride price may leave a woman vulnerable [18]. Socioeconomic vulnerability may make it difficult for individuals to negotiate condom use, for example in the context of transactional sex as observed in our study. While condoms are often provided free of charge within health facilities, there remain concerns regarding confidentiality, lack of privacy and judgmental attitudes towards youth, which deters them from accessing sexual health services [15]. Young men often reported accessing condoms from non-facility settings such as kiosks and pharmacies and in the context of high unemployment rates and other competing priorities, buying condoms for safer sex may not be a priority.

Overall, about half of young men surveyed reported being circumcised, well below the MoHCC target of 90% [10]. The majority had been circumcised in early adolescence, most likely through school programmes. Notably, a third of men had never been offered VMMC and a third of those who were offered it did not accept procedure, highlighting persisting *supply* and *demand* gaps. Among those who reported they would not take up VMMC even if offered, the main reasons were pain, irreversibility of the operation, and inability to work immediately after the procedure, consistent with other studies [19–21]. Possible initiatives to create demand include strengthening partnerships with the education sector to relay age-tailored accurate information, the use of peer support and role modelling or economic incentives [20–25]. Our findings revealed significant differences in the uptake of circumcision across the three different provinces with much higher rates in Bulawayo where cultural circumcision is more common, highlighting that the decision to get circumcised is a complex societally and culturally influenced process, and the need for programmes to engage in dialogue with communities including through faith-based groups [19, 21, 26, 27].

Even in those at substantial risk of HIV acquisition, awareness of PrEP was low. PrEP awareness was higher within the PrEP eligible group than in the overall group of youth surveyed. This higher awareness may be because the PrEP eligible group were more likely to have attended healthcare facilities (for example if they had received STI treatment or PEP), and perhaps seen PrEP promotional material there. Other high-risk groups such as sex workers are targeted for awareness campaigns [28–30]. The low levels of awareness in the wider group suggests that public awareness campaigns have been limited or ineffective. Awareness of pharmacological prophylaxis was higher in those who were more educated and wealthier, again highlighting social inequities. It is interesting to note that the 28 people acquiring PrEP outside of formal programmes were more often wealthier and educated, but not necessarily in an eligible population. It is unclear whether these people had other reasons for high-risk perception that were not picked up in our survey questions, for example they were men who have sex with men. Another possible explanation is that there is some demand for PrEP among youth and that those with wealth and education can bypass some of the barriers to access.

Notably, only 166 of the 6900 (2.4%) of youth in eligible groups reported both having heard of and been offered PrEP (Table 4). When offered, PrEP uptake was high, 60.7%, in the eligible population. Our results are concordant with 2020 census data for youth aged 15–24 years in Zimbabwe, the Combined HIV Adolescent PrEP and Prevention Study which included youth aged 13–24 years across South Africa, Uganda and Zimbabwe and the Malawi population-based HIV assessment [31–33]. All of these studies found that awareness of PrEP was low (8.1%, 25% and 15.0% respectively) and that willingness to take PrEP if offered was high (61.2%, 95% and 73.0% respectively) [31–33]. The Malawi population-based assessment, like our data, demonstrated that post secondary education and wealth quintile were associated with PrEP awareness [33]. Education and demand creation in target populations will be key if PrEP is to be utilized as part of an effective combination prevention strategy [28, 32, 34, 35]. Decentralised, peer-led and community-based PrEP delivery programmes for female sex workers in response to the COVID-19 pandemic resulted in a large increase in PrEP initiations in Zimbabwe [30]. PrEP marketing campaigns and youth friendly services have been suggested as methods to increase uptake of PrEP for adolescent girls and young women in Zimbabwe [34].

The majority (74.3%) of youth who had ever experienced forced sex in our cohort were not aware of PEP. Of concern, a small proportion (20.2%) of those who had experienced sexual violence in the last 12 months sought healthcare after the incident. Of those who did present to healthcare, only 36.0% were offered PEP. Possible explanations for the rest not being offered PEP include presentation more than 72 h after the incident, lack of PEP availability in the healthcare facility and lack of staff knowledge about PEP [36]. In 2018 in Zimbabwe, according to Zimbabwe National HIV and AIDS Strategic Plan for 2021–2025 (ZNASP IV), 400 cases of sexual assault were reported and referred for HIV testing. Only 25% of these presented within 72 h of the assault and only 15% received PEP [10]. In addition to low levels of community and healthcare provider awareness of PEP, addressing the specific social, personal, structural and economic barriers that youth face in accessing health services particularly sensitive services such as sexual assault or HIV prevention services, remains the paramount priority. Indeed, some of these barriers are compounded in those who are at the highest risk of HIV e.g. youth who are sex workers, men who have sex with men and transgender people [37].

Strengths of this study are the large randomly selected representative population across three provinces, the rigorous subgroup analysis and comprehensive assessment of multiple methods of HIV prevention simultaneously. Limitations include social desirability bias which may have led to under-reporting of sexual activity or risk behaviours, the study was also carried out in the context of a recent COVID-19 pandemic which may have limited access to services. PrEP and PEP efficacy is critically dependent on high adherence, but data on adherence to PrEP or completion of PEP courses was not collected which remains a significant limitation. We also did not collect data on certain characteristics that may increase risk of HIV acquisition. For example, male participants were not asked whether they have sex with men, an eligible group for PrEP, and specific questions about occupation were not asked which would have identified healthcare workers, a group that is at risk of occupational exposure to HIV who would be eligible for PEP.

## Conclusions

There continues to be low awareness and uptake of prevention interventions among youth in Zimbabwe. This is in a country which has experienced a long-standing severe generalized HIV epidemic, and which has had a longstanding investment in prevention programmes that led to a decline in HIV incidence among adults even before ART became widely available [38]. Our study highlights the importance of prioritizing youth, for programming that is age-appropriate and tailored to the needs of youth. Awareness of HIV prevention interventions remains a barrier to uptake. Innovative approaches, such as using social media and community youth advocates to enable reliable, age-appropriate information to reach youth need to be implemented. Health policy must address the persisting societal and structural equity barriers to access and uptake of effective prevention interventions if effective engagement of youth with healthcare services is to be achieved.

## Electronic supplementary material

Below is the link to the electronic supplementary material.

Supplementary Material 1

Supplementary Material 2

## Data Availability

The dataset is available on request from the corresponding author and subject to approval by the Medical Research Council of Zimbabwe.

## Abbreviations

ARV: Antiretroviral
CI: Confidence Interval
MoHCC: Ministry of Health and Child Care
OR: Odds Ratio
PEP: Post–exposure prophylaxis
PrEP: Pre–exposure prophylaxis
STI: Sexually transmitted infection
VMMC: Voluntary Medical Male Circumcision
ZNASP IV: Zimbabwe National HIV and AIDS Strategic Plan for 2021–2025

## References

[1] UNAIDS. UNAIDS Fact Sheet. - Global HIV statistics [Internet]. 2023 [cited 2024 Mar 28]. Available from: https://www.unaids.org/sites/default/files/media_asset/UNAIDS_FactSheet_en.pdf

[2] UNICEF, Adolescent HIV, prevention [Internet]. 2023 [cited 2024 Mar 28]. Available from: https://data.unicef.org/topic/hivaids/adolescents-young-people/#:~:text=Adolescents%20and%20young%20people%20represent,ages%20of%2010%20and%2019

[3] UNAIDS. Young people and HIV [Internet]. 2021 [cited 2024 Mar 28]. Available from: https://www.unaids.org/sites/default/files/media_asset/young-people-and-hiv_en.pdf

[4] Khalifa A, Stover J, Mahy M, Idele P, Porth T, Lwamba C. Demographic change and HIV epidemic projections to 2050 for adolescents and young people aged 15–24. Glob Health Action. 2019;12(1). 10.1080/16549716.2019.1662685 PMC6746261 31510887

[5] The Joint United Nations Programme on HIV/AIDS (UNAIDS). HIV prevention 2025 road map — Getting on track to end AIDS as a public health threat by 2030. Geneva; 2022.

[6] Clair-Sullivan NS, Mwamba C, Whetham J, Moore CB, Darking M, Vera J. Barriers to HIV care and adherence for young people living with HIV in Zambia and mHealth. Mhealth. 2019;5. 10.21037/mhealth.2019.09.02 PMC6789205 31620472

[7] Chikwari CD, Dringus S, Ferrand RA. Barriers to, and emerging strategies for, HIV testing among adolescents in sub-Saharan Africa. Vol. 13, Current Opinion in HIV and AIDS. 2018. 10.1097/COH.0000000000000452 29401121

[8] Pickles M, Gregson S, Moorhouse L, Dadirai T, Dzamatira F, Mandizvidza P et al. Strengthening the HIV prevention cascade to maximise epidemiological impact in eastern Zimbabwe: a modelling study. Lancet Glob Health. 2023;11(7). 10.1016/S2214-109X(23)00206-1 37349036

[9] Dziva Chikwari C, Dauya E, Bandason T, Tembo M, Mavodza C, Simms V, et al. The impact of community-based integrated HIV and sexual and reproductive health services for youth on population-level HIV viral load and sexually transmitted infections in Zimbabwe: protocol for the CHIEDZA cluster-randomised trial. Wellcome Open Res. 2022;7:54. 38162283 10.12688/wellcomeopenres.17530.2 PMC10755263

[10] Ministry of Health and Child Care Zimbabwe. National AIDS Council Zimbabwe. Zimbabwe National HIV and AIDS Strategic Plan 2021–2025. 2021.

[11] Chemaitelly H, Awad SF, Shelton JD, Abu-Raddad LJ. Sources of HIV incidence among stable couples in sub-Saharan Africa. J Int AIDS Soc. 2014;17. 10.7448/IAS.17.1.18765 PMC3935448 24560339

[12] Ferrand R, Dauya E, Dziva Chikwari C. Impact of integrated community-based HIV and sexual and reproductive health services for youth aged 16–24 years on population-level HIV outcomes in Zimbabwe: the CHIEDZA cluster randomized trial. 2024.

[13] Thoth CA, Tucker C, Leahy M, Stewart SM. Self-disclosure of serostatus by youth who are HIV-positive: A review. 37, J Behav Med. 2014. 10.1007/s10865-012-9485-2 23277232

[14] Jimu SE, Ntoimo LFC, Okonofua FE. Prevalence and determinants of condom use among the youth in Malawi: evidence from the 2015/16 Malawi demographic and health survey. Reprod Health. 2023;20(1). 10.1186/s12978-023-01714-9 PMC10664505 37990255

[15] Aventin Á, Gordon S, Laurenzi C, Rabie S, Tomlinson M, Lohan M et al. Adolescent condom use in Southern Africa: narrative systematic review and conceptual model of multilevel barriers and facilitators. BMC Public Health. 2021;21(1). 10.1186/s12889-021-11306-6 PMC8234649 34172027

[16] Worth D. Sexual Decision-Making and AIDS: why condom promotion among vulnerable women is likely to fail. Stud Fam Plann. 1989;20(6). 2623725

[17] Schoepf BG. Culture, sex research and AIDS prevention in Africa. In: Culture and Sexual Risk: Anthropological Perspectives on AIDS. 2004.

[18] Madiba S, Ngwenya N. Cultural practices, gender inequality and inconsistent condom use increase vulnerability to HIV infection: narratives from married and cohabiting women in rural communities in Mpumalanga Province, South Africa. Glob Health Action. 2017;10:sup2. 10.1080/16549716.2017.1341597 PMC5645648 28678650

[19] Kasprzyk D, Tshimanga M, Hamilton DT, Gorn GJ, Montaño DE. Identification of Key Beliefs Explaining Male Circumcision Motivation Among Adolescent Boys in Zimbabwe: Targets for Behavior Change Communication. AIDS Behav [Internet]. 2018 Feb 1 [cited 2024 Jan 24];22(2):454–70. Available from: https://pubmed.ncbi.nlm.nih.gov/28083832/10.1007/s10461-016-1664-7 28083832

[20] DeCelles J, Hershow RB, Kaufman ZA, Gannett KR, Kombandeya T, Chaibva C et al. Process evaluation of a sport-based voluntary medical male circumcision demand-creation intervention in Bulawayo, Zimbabwe. J Acquir Immune Defic Syndr (1988). 2016;72. 10.1097/QAI.0000000000001172 PMC5054959 27749598

[21] Hatzold K, Mavhu W, Jasi P, Chatora K, Cowan FM, Taruberekera N et al. Barriers and motivators to voluntary medical male circumcision uptake among different age groups of men in Zimbabwe: results from a mixed methods study. PLoS ONE. 2014;9(5). 10.1371/journal.pone.0085051 PMC4011705 24802746

[22] Carrasco MA, Grund JM, Davis SM, Ridzon R, Mattingly M, Wilkinson J et al. Systematic review of the effect of economic compensation and incentives on uptake of voluntary medical male circumcision among men in sub-Saharan Africa. AIDS Care [Internet]. 2018 Sep 2 [cited 2024 Jan 24];30(9):1071–82. Available from: https://pubmed.ncbi.nlm.nih.gov/29566546/10.1080/09540121.2018.1453921 PMC6800131 29566546

[23] Thirumurthy H, Masters SH, Rao S, Murray K, Prasad R, Zivin JG et al. The Effects of Providing Fixed Compensation and Lottery-Based Rewards on Uptake of Medical Male Circumcision in Kenya: A Randomized Trial. J Acquir Immune Defic Syndr [Internet]. 2016 Oct 1 [cited 2024 Jan 24];72 Suppl 4(Suppl 4):S309–15. Available from: https://pubmed.ncbi.nlm.nih.gov/27404012/10.1097/QAI.0000000000001045 PMC5054965 27404012

[24] Thomas R, Skovdal M, Galizzi MM, Schaefer R, Moorhouse L, Nyamukapa C et al. Improving risk perception and uptake of voluntary medical male circumcision with peer-education sessions and incentives, in Manicaland, East Zimbabwe: study protocol for a pilot randomised trial. Trials. 2020;21(1). 10.1186/s13063-020-4048-2 PMC6979356 31973744

[25] Kennedy CE, Yeh PT, Atkins K, Fonner VA, Sweat MD, O’Reilly KR et al. Economic compensation interventions to increase uptake of voluntary medical male circumcision for HIV prevention: A systematic review and meta-analysis. 15, PLoS ONE. 2020. 10.1371/journal.pone.0227623 PMC6961886 31940422

[26] Chikutsa A, Maharaj P. Social representations of male circumcision as prophylaxis against HIV/AIDS in Zimbabwe. BMC Public Health. 2015;15(1). 10.1186/s12889-015-1967-z PMC4489047 26133368

[27] Khumalo-Sakutukwa G, Lane T, van-Rooyen H, Chingono A, Humphries H, Timbe A et al. Understanding and addressing socio-cultural barriers to medical male circumcision in traditionally non-circumcising rural communities in sub-Saharan Africa. Cult Health Sex. 2013;15(9). 10.1080/13691058.2013.807519 PMC3810456 23815101

[28] Moorhouse L, Schaefer R, Thomas R, Nyamukapa C, Skovdal M, Hallett TB et al. Application of the HIV prevention cascade to identify, develop and evaluate interventions to improve use of prevention methods: examples from a study in East Zimbabwe. 22, J Int AIDS Soc. 2019. 10.1002/jia2.25309 PMC6643077 31328375

[29] Cowan FM, Chabata ST, Musemburi S, Fearon E, Davey C, Ndori-Mharadze T et al. Strengthening the scale-up and uptake of effective interventions for sex workers for population impact in Zimbabwe. J Int AIDS Soc. 2019;22(S4). 10.1002/jia2.25320 PMC6643097 31328445

[30] Matambanadzo P, Busza J, Mafaune H, Chinyanganya L, Machingura F, Ncube G, et al. It went through the roof: an observation study exploring the rise in PrEP uptake among Zimbabwean female sex workers in response to adaptations during Covid-19. J Int AIDS Soc. 2021;24:S6. 10.1002/jia2.25813 PMC8554216 34713613

[31] Ministry of Health and Child Care Zimbabwe. Zimbabwe Population-based HIV Impact Assessment 2020 (ZIMPHIA 2020). Harare; 2021.

[32] Dietrich J, Ahmed N, Nash S, Tshabalala G, Nematadzira T, Hornschuh S et al. A multi-country investigation of pre-exposure prophylaxis preferences among young people at risk of HIV in sub- Saharan Africa. J Int AIDS Soc [Internet]. 2021;24(SUPPL 1):27–8. Available from: http://ovidsp.ovid.com/ovidweb.cgi?T=JS%26PAGE=reference%26D=emed22%26NEWS=N%26AN=634542866

[33] Kabaghe AN, Singano V, Payne D, Maida A, Nyirenda R, Mirkovic K et al. Awareness of and willingness to use oral pre-exposure prophylaxis (PrEP) for HIV prevention among sexually active adults in Malawi: results from the 2020 Malawi population-based HIV impact assessment. BMC Infect Dis. 2023;23(1). 10.1186/s12879-023-08683-1 PMC10589952 37864140

[34] Skovdal M, Magoge-Mandizvidza P, Dzamatira F, Maswera R, Nyamukapa C, Thomas R et al. Improving access to pre-exposure prophylaxis for adolescent girls and young women: recommendations from healthcare providers in Eastern Zimbabwe. BMC Infect Dis. 2022;22(1). 10.1186/s12879-022-07376-5 PMC9035262 35461220

[35] Parmley L, Harris T, Chingombe I, Mapingure M, Mugurungi O, Rogers JH et al. Preexposure prophylaxis cascade among men who have sex with men in Zimbabwe. Top Antivir Med [Internet]. 2020;28(1):375. Available from: 10.1200/JCO.2020.38.29_suppl.994

[36] Tapesana S, Chirundu D, Shambira G, Gombe NT, Juru TP, Mufuta T. Clinical care given to victims of sexual assault at Kadoma general hospital, Zimbabwe: A secondary data analysis, 2016. BMC Infect Dis. 2017;17(1). 10.1186/s12879-017-2702-4 PMC5580318 28859613

[37] Govender K, Masebo WGB, Nyamaruze P, Cowden RG, Schunter BT, Bains A. HIV prevention in adolescents and young people in the Eastern and Southern African region: A review of key challenges impeding actions for an effective response. Open AIDS J. 2018;12(1). 10.2174/1874613601812010068 PMC6120102 30197723

[38] Gregson S, Garnett GP, Nyamukapa CA, Hallett TB, Lewis JJC, Mason PR et al. HIV decline associated with behavior change in eastern Zimbabwe. Science (1979). 2006;311(5761). 10.1126/science.1121054 16456081

