# Supplementary figures and images for: Awareness, access to and uptake of HIV prevention interventions among youth in Zimbabwe: a population-based survey
Source: BMC Infect Dis. 2025 May 16;25:709. doi: 10.1186/s12879-025-11076-1 (PMC12083137; doi:10.1186/s12879-025-11076-1)

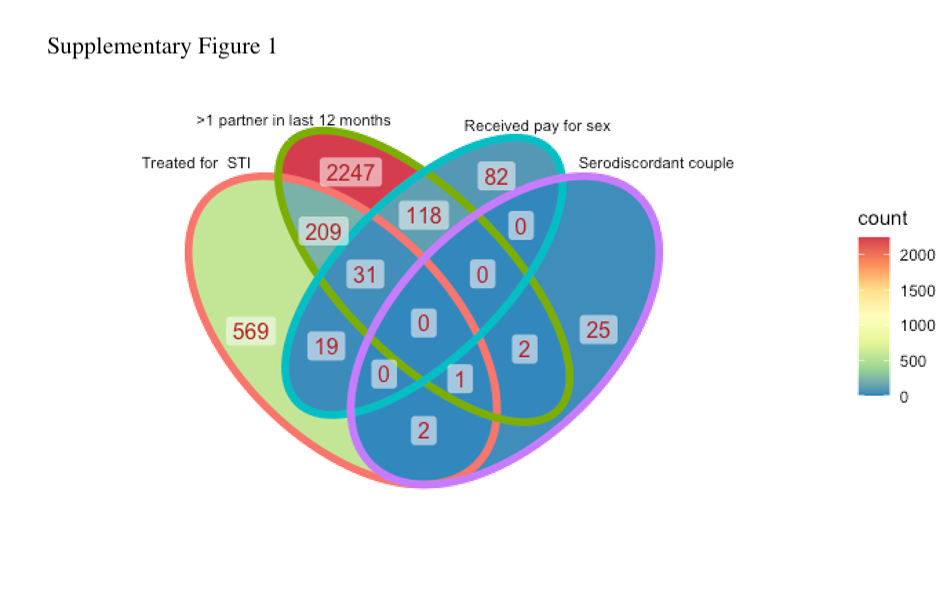

Supplement: Supplementary file 1 — Supplementary Material 1 [file 12879_2025_11076_MOESM1_ESM.tiff]
