# Supplementary material for: Awareness, access to and uptake of HIV prevention interventions among youth in Zimbabwe: a population-based survey
Source: BMC Infect Dis. 2025 May 16;25:709. doi: 10.1186/s12879-025-11076-1 (PMC12083137; doi:10.1186/s12879-025-11076-1)
Supplement: Supplementary file 2 — Supplementary Material 2 [file 12879_2025_11076_MOESM2_ESM.docx]

**Supplementary Table 1 –**Key survey questions asked to establish sexual behavioral characteristics

| **Survey Question** | **Response options** |
| --- | --- |
| Have you ever tested for HIV? | Yes  No  Don’t want to say |
| Can you tell us what was the result the LAST TIME you were tested for HIV? | Positive  Negative  I don’t know /Don’t want to say  I have never been tested for HIV |
| If you are married or living together as married or have a regular and steady sexual partner (even if you are not married or living as married), do you know your current partner’s HIV status? | Yes, s/he is HIV negative  Yes, s/he is HIV positive and s/he is not taking ART  Yes, s/he is HIV positive and I don’t know if s/he is taking ART  Yes, s/he is HIV positive and I know s/he is taking ART  I don’t know his/her HIV status  Don’t want to say  Not applicable, not married or living as married or no regular sexual partner |
| How old were you when you first had penetrative sexual intercourse, if ever? By this I mean when a man/husband/boyfriend/partner put his penis inside a woman’s vagina (vaginal sex), or inside a woman or a man’s anus or backside (anal sex). Sex can be between two people of the same gender and can have occurred even if someone was not sure they wanted it. If reports NEVER, probe: At what age did someone first have sexual intercourse with you, if ever? |  Record age in years  I can’t remember the age when I first had penetrative sex  Don’t want to say  I have not ever had penetrative sex |
| In your lifetime, how many different sexual partners have you had (including regular partner if applicable)?  Sexual partner refers to someone whom you have vaginal or anal sex with |  00 if none  Don’t want to say |
| In the past 12 months, with how many people have you had sex |  00 if none  Don’t want to say |
| People enter into sexual relations / have sex (vaginal or anal sex) for many different reasons. In the last 12 months, have you had sex or been sexually involved with anyone because he or she gave you or told you he or she would give you material support of any kind? By material support, we mean gifts, financial support for rent/school fees or cash or anything else | Yes  No  Don’t want to say  Not applicable, I have never had sex |
| In the last 12 months, have you provided someone (including sex workers but not necessarily only sex workers ) with money, or help to pay for their expenses, or do them a favour mainly to enter into sexual relations / have sex with them, or to keep having sex with them? | Yes  No  Don’t want to say  Not applicable, I have never had sex |
| Thinking about the last 12 months, how often have you used condoms when you had (vaginal or anal) sex? | Most of the times  Sometimes (about half the time)  Rarely  Never  Don’t want to say  Did not have sex in the last 12 months |
| Did you use a condom the LAST time you had sex? | Yes  No  Don’t want to say  I have never had sex |
| Where do you commonly get male condoms from or where would you go to get them, if you needed to use them? | CHIEDZA service  General government hospital/clinic or health centre  HIV / OI clinic*  ZNFPC clinic/centre  Private hospital clinic  Non-governmental organisation [Specify] ____  Pharmacy/Chemist  General dealer or shop/supermarket/kiosk  Street vendor  Bottle store/Bar  Public Toilets  Other public places e.g. bars, bathrooms, hotels  Family member or friend  Youth Centre  Church  Workplace  Other [Specify]____ |
| What factors make it difficult to get male condoms if you wanted to use them?  Read through all options and Tick all that apply | High cost  Poor quality  Embarrassed to go/ask  Lack of privacy/confidentiality in places where I would go for condoms  Limited opening hours of places where I would go for condoms  Distance/travel difficulties to places where I would go for condoms  Stockouts in places where I would go for condoms  Other [Specify]___ |
| Have you ever been treated for a sexually transmitted infection (STI). This includes discharge from your penis or vagina, warts, sores on private parts and infections such as chlamydia, gonorrhoea, syphilis, herpes. Excludes HIV). If multiple treatments, respond for when last treated for an STI | Yes, in the last year  Yes, more than a year ago  No  Don’t want to say |
| Have you ever been tested for STIs excluding HIV? This means having had a test for an STI such as gonorrhoea, chlamydia, herpes, HPV, syphilis, trichomonas vaginalis whether you had symptoms or not? | Yes, at CHIEDZA  Yes, elsewhere [Specify where]__  No  Don’t want to say |
| Have you ever heard of drugs that can be taken to prevent HIV infection AFTER possible exposure to the virus? (Post-exposure prophylaxis or PEP)  *When someone is exposed to HIV, e.g. the condom breaks or they have unprotected sex or a needlestick injury, a course of ART or ARV pills for one month can prevent HIV. This is called PEP.* | Yes  No |
| Have you ever taken PEP? | Yes  No  Don’t want to say  I have heard about PEP but haven’t taken it because I am HIV-positive  I have never heard about PEP until today |
| Have you ever heard of drugs that can be taken to prevent HIV infection BEFORE possible exposure to the virus? (Pre-exposure prophylaxis or PREP)  *When someone does not have HIV taking a pill on an ongoing basis to prevent them from getting HIV. This is called PREP. Most people who use PREP take it everyday. It needs to be taken before sex for it to work.* | Yes  No |
| Have you ever been offered PREP by a clinic or any other organisation? | Yes  No  Don’t want to say  I have never heard about PREP until today |
| Have you ever taken PREP? | Yes  No  Don’t want to say  I have never heard about PREP until today |
| MALES ONLY  Have you ever been offered / referred for voluntary male medical circumcision (VMMC) (e.g. at school, by a clinic, a VMMC programme/other?) | Yes  No |
| Who offered/referred you for VMMC? | CHIEDZA service  Offered at school  Offered at primary care clinic  Offered by NGO [Specify] ____  Other [Specify] ___  Never been offered/ referred for VMMC  7.23 Have you been circumcised?  Yes  No  Don’t want to say |
| At what age were you circumcised? |  years  Not applicable, I am not circumcised |
| Why did you get circumcised?  Allow participant to volunteer responses and Tick all that apply | For cultural reasons/ Traditional circumcision  HIV / STI protection (for myself)  HIV/ STI protection (for my partner(s))  Circumcision is a method of HIV prevention I can control  More able to attract partners (because of lower risk of transmitting HIV/STIs)  Penis would look better / cleaner  Improvement of my sexual pleasure  Improvement of my partner’s sexual pleasure  Able to have an erection for longer  Other [Specify]___  Not applicable, I am not circumcised |
| Why have you not been circumcised?  Allow participant to volunteer responses and Tick all that apply | I have never been offered circumcision  I am HIV positive so not suitable for me  Don’t know where to go to get circumcision  It’s too far to go to a place where I can get circumcision  Inconvenient opening hours  I don’t want to expose myself to female health providers  Lack of privacy and confidentiality in health facilities  I am worried about not being able to work after the procedure  Worried about the pain  Operation cannot be reversed  Risk of getting an infection  May affect my fertility  May affect my sexual pleasure or my sexual performance  Would make me feel less manly  I am using another HIV prevention method ([Specify method] ____  I abstain from sex / am faithful to one trusted partner  I don’t know what happens to my removed foreskin  Other (specify) ___  Don’t want to say  Not applicable, I am circumcised |
| If you wanted to get VMMC, do you know a place where you can easily have it done? | Yes  No  Not applicable, I am circumcised |
| How likely are you to get VMMC if the service is freely and easily accessible | Definitely  Probably  No opinion  Probably not  Definitely not  I am already circumcised |
| Sometimes people have sex even though they may not want to, either through being physically forced, or because they feel afraid of what they might do if they refuse. Have you ever been forced to have sex when you didn’t want to? This can include from a current/former husband/wife, boyfriend/girlfriend, partner, or a member of your family | Yes  No  Don’t want to say |
| In the past 12 months, how many times have you been forced to have sex when you did not want to? | Often  Sometimes  Once  I haven’t been forced to have sex in the past 12 months  Don’t want to say |
| After the unwanted sexual experience in the last 12 months, did you seek any healthcare services to protect yourself against HIV or other infections? | Yes  No  Don’t want to say  I have not been forced to have sex in the past months |
| Where did you access these healthcare services? | Primary health clinic or health centre  Hospital (including being admitted)  Special Rape/sexual assault clinic  Other [Specify]__  I have not been forced to have sex in the past months  Don’t want to say |
| What services were you offered at this health facility? | Read through all options and Tick all that apply  STI testing  STI treatment  HIV testing services  Counselling services  PEP  Emergency contraception  Referral to legal services  Referred to police  Participation in support groups  Referral to post-violence services  Transport voucher to access services  Other [Specify]_  Don’t know  Don’t want to say |
| What contraceptive method(s) are you (or your partner) currently using? | Male condom  Female condom  Oral contraceptive pills (daily)  Injection (Depo)  Implants (Jadelle/Norplant)  IUD/Loop  Diaphragm or ring  Foam/jelly  Male sterilisation  Female sterilisation  Breastfeeding / Lactational amenorrhoea method  Rhythm method/Safe days (avoiding sex on days I am fertile)  Withdrawal method (partner withdraws before ejaculation)  Other Traditional method [Specify]__  Other modern method [Specify]__  Don’t want to say  Not using contraception to prevent pregnancy  Not applicable, am not sexually active |
| What contraceptive method(s) have you (or your partner) used in the past 12 months? | Read through all options and Tick all that apply  Male condom  Female condom  Oral contraceptive pills (daily)  Injection (Depo)  Implants (Jadelle/Norplant)  IUD/Loop  Diaphragm or ring  Foam/jelly  Male sterilisation  Female sterilisation  Breastfeeding / Lactational amenorrhoea method  Rhythm method/Safe days (avoiding sex on days I am fertile)  Withdrawal method (partner withdraws before ejaculation)  Other Traditional method [Specify]_______  Other modern method [Specify]____  Don’t want to say  Not using contraception to prevent pregnancy  Not applicable, am not sexually active |
| Have you ever been pregnant (excluding current pregnancy)? Include pregnancies that did not go to full term (i.e. miscarriage, termination of pregnancy/result in birth? | Yes  No |

**Supplementary table 2:** Demographic and sexual behavioral characteristics of those with missing response to one or more of the sexual behavioral characteristics^1^ compared to the population with no missing responses in these variables

| **Characteristic** | **Number missing** | **No missing sexual behavioral characteristic^1^**  **N = 17091 (97%)^2^** | **Any missing sexual behavioral characteristic**  **N = 592 (3.3%)** | **p-value^3^** |
| --- | --- | --- | --- | --- |
| Sex | 1 |  |  | <0.001 |
| Female |  | 10,515 (60.0%) | 227 (36.1%) |  |
| Male |  | 6,575 (40.0%) | 365 (63.9%) |  |
| Age (years) | 0 | 20 (18.0, 22.0) | 22 (20.0, 23.0) | <0.001 |
| Province | 0 |  |  | <0.001 |
| Bulawayo |  | 5,652 (34.1%) | 317 (57.1%) |  |
| Harare |  | 5,691 (17.5%) | 158 (14.5%) |  |
| Mashonaland East |  | 5,748 (48.3%) | 117 (28.4%) |  |
| Maximum level of education | 0 |  |  | 0.003 |
| Primary school |  | 3,166 (19.3%) | 88 (15.8%) |  |
| Secondary school |  | 12,585 (73.4%) | 432 (73.1%) |  |
| Post secondary |  | 1,340 (7.2%) | 72 (11.1%) |  |
| Currently in formal employment or education | 0 |  |  | 0.5 |
| Yes |  | 5,619 (32.1%) | 180 (30.2%) |  |
| No |  | 11,472 (67.9%) | 412 (69.8%) |  |
| Ever had sexual intercourse | 89 |  |  | <0.001 |
| Yes |  | 10,827 (62.7%) | 466 (94.4%) |  |
| No |  | 6,264 (37.3%) | 37 (5.6%) |  |
| Age of first sexual intercourse (years) | 256 | 18 (16.0, 19.0) | 17 (16.0, 18.0) | <0.001 |
| Marital Status | 0 |  |  | <0.001 |
| Yes |  | 3,505 (20.3%) | 54 (8.5%) |  |
| No |  | 13,586 (79.7%) | 538 (91.5%) |  |
| Number of lifetime partners | 480 | 1 (0.0, 3.0) | 1 (0.0, 3.0) | 0.056 |
| More than one lifetime partner | 480 |  |  | 0.015 |
| Yes |  | 6,462 (37.9%) | 53 (50.0%) |  |
| No |  | 10,629 (62.1%) | 59 (50.0%) |  |
| More than one partner in last 12 months | 228 |  |  | <0.001 |
| Yes |  | 2,423 (14.0%) | 185 (50.8%) |  |
| No |  | 14,668 (86.0%) | 179 (49.2%) |  |
| Number of partners in last 12 months | 228 | 1 (0.0, 1.0) | 2 (1.0, 3.0) | <0.001 |
| Awareness of partner's HIV status | 190 |  |  | <0.001 |
| Known |  | 4,482 (26.5%) | 141 (25.0%) |  |
| Unknown |  | 3,493 (20.5%) | 192 (36.8%) |  |
| No current regular sexual partner |  | 2,705 (15.3%) | 179 (33.2%) |  |
| Never had intercourse |  | 6,264 (37.6%) | 37 (5.1%) |  |
| Ever had an HIV test | 46 |  |  | 0.009 |
| Yes |  | 11,722 (67.7%) | 432 (78.2%) |  |
| No |  | 5,369 (32.3%) | 114 (21.8%) |  |
| Had an HIV test and know the results | 5,529 |  |  | <0.001 |
| Yes |  | 11,592 (98.8%) | 411 (94.5%) |  |
| No |  | 130 (1.2%) | 21 (5.5%) |  |
| Ever tested for an STI^4^ (not HIV) | 48 |  |  | 0.005 |
| Yes |  | 1,916 (9.8%) | 86 (14.6%) |  |
| No |  | 15,175 (90.2%) | 458 (85.4%) |  |
| Ever treated for an STI^4^ | 108 |  |  | <0.001 |
| Yes |  | 783 (4.2%) | 48 (9.8%) |  |
| No |  | 16,308 (95.8%) | 436 (90.2%) |  |
| HIV negative in a serodiscordant relationship | 0 |  |  | <0.001 |
| Yes |  | 30 (0.2%) | 0 (0.0%) |  |
| No |  | 13,089 (76.6%) | 344 (56.4%) |  |
| Unknown status of participant or partner |  | 3,972 (23.3%) | 248 (43.6%) |  |
| Known HIV positive status | 0 |  |  | 0.13 |
| Yes |  | 410 (2.4%) | 25 (3.8%) |  |
| No |  | 16,681 (97.6%) | 567 (96.2%) |  |
| Ever received payment or goods for sex | 240 |  |  | <0.001 |
| Yes |  | 235 (1.4%) | 15 (5.0%) |  |
| No |  | 16,856 (98.6%) | 337 (95.0%) |  |
| Ever paid for sex | 240 |  |  | <0.001 |
| Yes |  | 181 (1.1%) | 14 (4.2%) |  |
| No |  | 16,910 (98.9%) | 338 (95.8%) |  |
| Ever been forced to have sex | 72 |  |  | 0.014 |
| Yes |  | 257 (1.4%) | 15 (2.9%) |  |
| No |  | 16,787 (98.6%) | 552 (97.1%) |  |
| Ever used any recreational drugs | 49 |  |  | <0.001 |
| Yes |  | 1,037 (6.0%) | 112 (20.9%) |  |
| No |  | 16,029 (94.0%) | 456 (79.1%) |  |
| Ever injected drugs | 55 |  |  | 0.083 |
| Yes |  | 7 (0.0%) | 1 (0.3%) |  |
| No |  | 17,053 (100.0%) | 567 (99.7%) |  |
| High Alcohol Use^5^ | 0 |  |  | <0.001 |
| Yes |  | 986 (5.6%) | 100 (16.1%) |  |
| No |  | 16,105 (94.4%) | 492 (83.9%) |  |
| Ever taken PEP^6^ | 59 |  |  | <0.001 |
| Yes |  | 89 (0.4%) | 22 (3.1%) |  |
| No |  | 17,002 (99.6%) | 511 (96.9%) |  |

1. Missing data in for any one of the following questions:

“ever had an HIV test”, “ever had an STI test”, “ever had sti treatment”, “ever had intercourse”, “number of partners in last 12 months”, “number of lifetime partners”, “ever paid for sex”, “ever received payment or goods for sex”, “ever heard of PrEP”, “Ever heard of PEP”, “Ever taken PEP”

1. All percentages weighted for clustering
2. Chi-squared test with Rao & Scott's second-order correction
3. Sexually Transmitted Infection
4. Alcohol use disorders identification test (AUDIT) score >12
5. HIV Post Exposure Prophylaxis

**Supplementary table 3:** Associations between sociodemographic factors and regular condom use in a multivariable mixed effects models adjusted for the variables shown as fixed effects, cluster and province as random effects.

| Independent Variable | | **OR [95% CI]**^1^ | **p values (Wald Test)** |  |
| --- | --- | --- | --- | --- |
| Age Group | |  |  |  |
| 18-20 | | — |  |  |
| 21-24 | | 0.93 [0.85-1.03] | 0.112 |  |
| Sex (Female vs Male) | | 0.42 [0.38-0.47] | <0.001 |  |
| Wealth Quintile | |  | 0.001 |  |
| Poorest | | — |  |  |
| Poor | | 0.96 [0.82- 1.13] |  |  |
| Medium | | 1.07 [0.91- 1.26] |  |  |
| Rich | | 1.25 [1.06 - 1.48] |  |  |
| Richest | | 1.19 [1.00- 1.41] |  |  |
| Maximum level of education | |  | 0.006 |  |
| Primary school | | — |  |  |
| Secondary school | | 1.19 [1.05-1.36] |  |  |
| Post-secondary | | 1.24 [1.01-1.52]  [1.01- 1.52] |  |  |
| Married or living as married   vs unmarried | | 0.09 [0.08-0.11] | <0.001 |  |
|  | ^1^OR = Odds Ratio, CI = Confidence Interval | | | |

**Supplementary table 4:** Location where condoms were purchased, by sex (weighted percentages)

| **Reported location of Purchase** | **Female, N = 3271** | **Male, N = 3304** | **p-value^2^** |  |
| --- | --- | --- | --- | --- |
| Pharmacy/Chemist | 1,137 (32.6%) | 1,169 (33.6%) | 0.5 |  |
| General dealer or shop/supermarket/kiosk | 902 (27.0%) | 1,217 (36.7%) | <0.001 |  |
| General government hospital/clinic  or health centre | 918 (30.1%) | 1,060 (31.8%) | 0.3 |  |
| Family member or friend | 204 (6.6%) | 689 (20.9%) | <0.001 |  |
| CHIEDZA service | 268 (8.0%) | 378 (10.4%) | 0.071 |  |
| Bottle store/Bar | 124 (3.8%) | 453 (13.6%) | <0.001 |  |
| Street vendor | 106 (3.6%) | 260 (7.6%) | <0.001 |  |
| HIV / Opportunistic infection (OI) clinic | 137 (4.0%) | 152 (4.1%) | 0.8 |  |
| Other public places e.g. bars, bathrooms, hotels | 51 (1.5%) | 193 (6.0%) | <0.001 |  |
| Public Toilets | 62 (2.1%) | 149 (4.6%) | <0.001 |  |
| Private hospital clinic | 90 (2.4%) | 102 (3.1%) | 0.2 |  |
| Youth Centre | 62 (1.9%) | 98 (2.9%) | 0.036 |  |
| Non-governmental organisation | 48 (1.6%) | 53 (1.5%) | 0.6 |  |
| Workplace | 26 (0.7%) | 62 (1.8%) | 0.005 |  |
| ZNFPC clinic/centre | 46 (1.5%) | 39 (1.1%) | 0.3 |  |
| Church | 2 (0.1%) | 6 (0.1%) | 0.3 |  |
| Other | 148 (4.3%) | 55 (1.5%) | <0.001 |  |
|  | | | | |
| ^1^chi-squared test with Rao & Scott's second-order correction | | | | |

**Supplementary Table 5**: Reported Barriers to buying condoms (weighted percentages)

| **Reported Barrier** | **Female, N = 2503** | **Male, N = 1922** | **p-value^1^** |  |
| --- | --- | --- | --- | --- |
| Embarrassed to go/ask | 1,602 (65.4%) | 942 (50.2%) | <0.001 |  |
| Lack of privacy/confidentiality  in places where I would go for condoms | 1,163 (46.6%) | 827 (41.7%) | 0.046 |  |
| High cost | 502 (21.1%) | 671 (36.2%) | <0.001 |  |
| Poor quality | 460 (19.7%) | 523 (26.9%) | 0.011 |  |
| Limited opening hours of places where I would go for condoms | 186 (8.0%) | 207 (11.5%) | 0.003 |  |
| Distance/travel difficulties to places where I would go for condoms | 174 (7.8%) | 181 (11.1%) | 0.013 |  |
| Stockouts in places where I would go for condoms | 166 (7.7%) | 250 (13.0%) | 0.004 |  |
| Other | 243 (9.1%) | 79 (4.2%) | <0.001 |  |
| ^1^chi-squared test with Rao & Scotts second-order correction | | | | |

**Supplementary table 6:** Reported reasons for circumcision in men who are circumcised (weighted percentages)

| Reason for listed for circumcision | Overall, N = 3369 (100%) |
| --- | --- |
| For HIV/STI protection (for myself) | 2,521 (75.6%) |
| For HIV/STI protection (for others) | 1,470 (43.5%) |
| Penis would look better / cleaner | 1,044 (30.0%) |
| Circumcision is a method of HIV prevention I can control | 867 (25.4%) |
| Improvement of my sexual pleasure | 412 (11.7%) |
| For cultural reasons | 322 (9.1%) |
| More able to attract partners.  (because of lower risk of transmitting HIV/STIs) | 286 (8.1%) |
|  |  |
| Improvement of my partner’s sexual pleasure | 256 (7.1%) |
| Able to have an erection for longer | 229 (6.7%) |
| Other reason | 199 (5.3%) |

**Supplementary table 7:** Reported reasons for not being circumcised in uncircumcised men (weighted percentages)

| **Reason listed for not being circumcised** | **Overall, N = 3410**  **(100%)^1^** |
| --- | --- |
| Worried about pain | 1,523 (44.5%) |
| Operation cannot be reversed | 351 (9.8%) |
| Never been offered circumcision | 299 (9.2%) |
| Worried about not being able to work after circumcision | 314 (9.0%) |
| May affect sexual pleasure or sexual performance | 247 (7.4%) |
| I don't know what happens to my removed foreskin | 202 (5.5%) |
| Risk of getting an infection | 183 (5.2%) |
| I abstain from sex / Am faithful to one trusted partner | 112 (3.4%) |
| May affect fertility | 106 (3.1%) |
| Would make me feel less manly | 117 (3.1%) |
| Lack of privacy and confidentiality in healthcare settings | 64 (2.1%) |
| Inconvenient opening hours | 71 (2.0%) |
| Concern about exposing self to female healthcare providers | 67 (1.8%) |
| Don't know where to get circumcision | 55 (1.7%) |
| It's too far to get to a place to get circumcision | 53 (1.6%) |
| Living with HIV, so not suitable | 47 (1.4%) |
| Using another HIV prevention method | 34 (1.0%) |
| Other reason | 487 (13.1%) |
|  |  |
